# Cyrus S. Knapp

**Published:** 1873-11

**Authors:** 


### OBITUARY.

*Editor American Journal of Dental Science:*

On the 3d day of July, 1873, at a meeting of the New Orleans Dental Association the following preamble and resolutions were passed:

Whereas Divine Providence, in His allwise dispensation, has seen fit to allow the laws of nature to be exercised in removing from life's busy scenes and cares the very estimable gentleman Cyrus S. Knapp, D. D. S., at his residence, near Jackson, Mississippi, long before having attained that full measure of earthly existence which has been allotted to many ; and

Whereas, in his demise society has been deprived of a useful worthy member ; his wife of a loving devoted hnsband ; his children and brothers of an affectionate father and brother ; whilst his numerous friends and relations have sustained the irreparable loss of a warm and genial Friend ; the Dental Profession of one of its highest ornaments ; and this Association of one of its corresponding members ;

*Therefore be it Resolved,* That we tender to his family and relations our heart-felt sympathy and condolence for their sad bereavement, and that this preamble and resolution be spread upon the minutes of this meeting, and that a copy of the same be forwarded to his grief-stricken family, and that copies likewise be furnished to the Dental Journals for publication.

